# Analysis of a possible independent origin of triploid *P. formosa *outside of the Río Purificación river system

**DOI:** 10.1186/1742-9994-4-13

**Published:** 2007-05-15

**Authors:** Susanne Schories, Kathrin P Lampert, Dunja K Lamatsch, Francisco J García de León, Manfred Schartl

**Affiliations:** 1 Physiological Chemistry I, Biocenter, University of Würzburg, Biozentrum, Am Hubland, D-97074 Würzburg, Germany; 2 Department of Animal and Plant Sciences, The University of Sheffield, Sheffield S10 2TN, UK; 3 Centro de Investigaciones Biológicas del Noroeste, S.C. (CIBNOR, S.C.), Mar Bermejo No. 195, Col. Playa Palo de Santa Rita, La Paz, BCS, 23090, México

## Abstract

**Background:**

Unisexuality, or all female reproduction, is rare among vertebrates. Studying these exceptional organisms may give useful information with respect to the evolution and maintenance of sexual reproduction. *Poecilia formosa *was the first unisexual vertebrate species to be detected and since then has served as a paradigmatic organism for unisexuality and studies on the evolution of sex. It reproduces through gynogenesis, using sperm of males from related species to trigger parthenogenetic development of the unreduced diploid eggs. Like in other unisexual vertebrates, triploids occur in a certain range of *P. formosa*. It has been suggested that the addition of the host species derived third chromosome set is evolutionary important. Clonal organisms lack sufficient genotypic diversity for adaptive changes to variable environments. Also non-recombining genomes cannot purge deleterious mutations and therefore unisexual organisms should suffer from a genomic decay. Thus, polyploidization leading to triploidy should bring "fresh" genetic material into the asexual lineage. To evaluate the importance of triploidy for maintaining the asexual species, it is important to know whether such an introgression event happens at a reasonable frequency.

**Results:**

In an earlier study it was found that all triploid *P. formosa *in the Rio Purificación river system are of monophyletic origin. Here we have analyzed fish from a different river system. Using microsatellite analysis we can show that the triploids from this new location are genetically divergent and most probably of an independent origin.

**Conclusion:**

Our data support the hypothesis that triploidy was not a single chance event in the evolutionary history of *P. formosa *and hence might be a relevant mechanism to increase genotypic divergence and at least partially counteract the genetic degeneration connected to asexuality. It is, however, much rarer than in other asexual vertebrates analyzed so far and thus probably only of moderate evolutionary importance for the maintenance of the asexual breeding complex.

## Background

*Poecilia formosa *is an all female teleost fish species. In fact it was the first asexual vertebrate to be described [1] and has been a paradigmatic organism for studies on the evolution of sexual reproduction and the maintenance of asexuality [2-4]. Its mode of reproduction is gynogenesis, a form of parthenogenesis, in which the embryonic development of unreduced diploid eggs is triggered by sperm from males of closely related species.

Usually the DNA of the sperm does not reach the egg nucleus resulting in clonal offspring. In exceptional cases the exclusion mechanism fails and either small fragments of single chromosomes, designated supernumerary chromosomes or microchromosomes [5], or the full chromosome set of the whole haploid genome of the sperm is included [6-8].

Fish with additional microchromosomes readily can form new clones that stably transmit the microchromosome. This phenomenon has been found in laboratory broods as well as in wild populations and occurs obviously at a reasonable frequency [9]. Interestingly, microchromosome carrying fish have been reported only from the Río Purificación [10].

The situation for the triploids is different: all triploids that occurred in the laboratory so far were sterile, while triploids collected in nature produce all triploid offspring [8,11]. Those triploids have been found in the Río Purificación and its tributaries [12,13], as well as in the hydrologically connected Río Soto la Marina river system. Apart from that only in the river system adjacent to the south, the Río Guayalejo, some stable triploid populations exist [6]. At the periphery of the range of *P. formosa*, triploids appear to be extremely rare. Enigmatic reports exist from the Río Grande (or Río Bravo) basin where during steady large-scale sampling (N = 5000) only four triploids were collected in ten years. Another single triploid was found in the Río Tuxpan system. The rare triploids outside the upper Río Purificación were interpreted to be "sterile first generation triploids" like the laboratory born [13]. Triploids are frequent where *P. formosa *is found together with *P. limantouri*, which serves as sperm donor, but absent or rare where *P. formosa *is sympatric with the alternative sperm donors *P. mexicana *or *P. latipinna*. The hypothesis was put forward that triploids may arise de-novo throughout the whole range, but are only fertile and can survive only in the region, where *P. formosa *occurs together with *P. limantouri *[14], namely in the Ríos Purificación, Soto la Marina and Guayalejo. An alternative hypothesis, however, is that reproductive triploidy is an extremely rare event, which occurred so far only once in an area, where *P. formosa *mates with *P. limantouri*. From there the triploid fish spread and invaded the neighboring river systems but have not reached the periphery of the range.

A microsatellite based study on fish from the Río Purificación suggested that all triploids that were analyzed belonged to only two clonal lineages and had a monophyletic origin [12]. This stands in contrasts to the diploids, which display a high number of different clones [12,15]. It led to the conclusion that the generation of a fertile triploid *P. formosa *is a rare, if not a unique event.

We report here a molecular genetic analysis of a stable population of triploid *P. formosa *outside the Río Purificación system. The allelic and genotypic variability of the triploid lineages and the question how recently they might have emerged was studied. Phylogenetic analysis revealed that the triploid clones from the new locality most probably are of independent origin of the Río Purificación clones. Thus fertile triploids may have been generated at least twice, but this event is much less frequent contrary to other asexual vertebrates.

## Results

In a genetic survey of natural populations of *P. formosa *we analyzed 114 and 100 animals collected in 2002 and 2005, respectively, at a site in the Río Guayalejo (G) river system. This river system is the next south but is hydrogeographically separated from the Río Purificación and Río Soto la Marina system (P). Flow cytometric measurements of DNA content revealed that 14 individuals of the 2002 (12%) and 16 individuals of the 2005 sample (16%) were triploids.

The eleven microsatellite loci used to genotype the triploid clones detected very different levels of variability (Table 1). Combining all loci the allelic diversity was higher in triploids from Río Purificación (P) than in triploids from Río Guayalejo (G) (Table 1). Triploids from both sites shared 35% of all alleles found in triploid individuals.

**Table 1:** Variability of microsatellite loci in different sites (G – Río Guayalejo; P – Río Purificación) and years.

	site – year					
Locus	G – 2002	G – 2005	P – 2002	P – 2005	total	% shared alleles

N	11	15	26	13	65	
mCA20	2	2	16	12	17	11.76
MSD23	6	6	9	9	14	42.86
Sat1	3	3	5	5	8	12.5
KonD15	3	3	5	5	6	33.33
PR39	3	3	4	4	5	40
mCA16	2	2	3	3	3	66.67
MATG31	3	3	3	3	4	50
MATG38	3	3	4	4	4	75
MATG44	2	2	3	3	3	66.67
MATG61	3	3	3	3	4	50
MATG78	2	2	2	2	3	33.33

Given are numbers of alleles for each locus and in total for all triploids found. Only individuals that could be genotyped at all 11 loci were taken into account. The percentages of shared alleles between sites for each locus are given.

In a previous study on the triploids from the Río Purificación system the triploid genotypes were separated in two lineages based on their overall genotypic differences in the three microsatellite loci used [12]. The lineages were designated Y and Z. Following this nomenclature the new lineage from G on the basis of the same three microsatellite loci was named X. When additional microsatellite loci were analyzed in this study they revealed that each of the three lineages includes several clonal genotypes. The clonal variation between individual genotypes was mainly due to the variation at the two most variable loci mCA20 and MSD23. The overall genotypic variation was lower in G (4 different genotypes) than in P (20 different genotypes) (Table 2). The recapture rate of clones was also higher in G: three of the four clones found in 2002 could be found again in 2005, while in P of the 15 clones found in 2002 only three were found again in 2005. In and between the years none of the triploid clones found in one site could be found in the other site (Table 2). The lower genotypic variability in G was confirmed by a frequency analysis according to Menken et al. [16]: the proportion of distinguishable clones in G (0.28 (mean of both years)) was lower than in P (0.60 (mean of both years)). The clonal diversity value (mean of both years) was also lower in G (0.57) than in P (0.88) (Table 3).

**Table 2:** Genotypes of all triploids found at the two sites

				**genotypes**										
	**#**	**site**	**year**	**mCA20**	**MSD 23**	**Sat1**	**KonD15**	**PR39**	**mCA16**	**mATG31**	**mATG38**	**mATG44**	**mATG61**	**mATG78**

**X1**	1	**G**	2002	115-115-**196**	**205**-217-245	**102**-**124**-**152**	255-280-**282**	141-**153**-166	203-203-221	**70**-73-79	134-170-185	115-115-140	133-**142**-145	**192**-198
**X2**	7	**G**	2002/2005	115-115-**196**	**205**-**229**-241	**102**-**124**-**152**	255-280-**282**	141-**153**-166	203-203-221	**70**-73-79	134-170-185	115-115-140	133-**142**-145	**192**-198
**X3**	15	**G**	2002/2005	115-115-**196**	**205**-**229**-245	**102**-**124**-**152**	255-280-**282**	141-**153**-166	203-203-221	**70**-73-79	134-170-185	115-115-140	133-**142**-145	**192**-198
**X4**	3	**G**	2002/2005	115-115-**196**	**205**-233-245	**102**-**124**-**152**	255-280-**282**	141-**153**-166	203-203-221	**70**-73-79	134-170-185	115-115-140	133-**142**-145	**192**-198
**Y1**	1	**P**	2002	115-115-208	201-217-241	116-122-144	255-258-270	141-147-166	191-203-221	73-76-79	134-170-185	115-118-140	133-139-145	198-201
**Y2**	2	**P**	2002	115-115-252	205-217-245	116-122-144	255-258-270	141-147-166	191-203-221	73-76-79	134-170-185	115-118-140	133-139-145	198-201
**Y3**	2	**P**	2002	115-115-258	205-217-253	116-122-144	255-258-270	141-147-166	191-203-221	73-76-79	134-170-185	115-118-140	133-139-145	198-201
**Y4**	4	**P**	2002/2005	115-115-236	209-213-245	116-122-144	255-258-270	141-147-166	191-203-221	73-76-79	134-170-185	115-118-140	133-139-145	198-201
**Y5**	1	**P**	2002	115-115-240	209-213-245	116-122-144	255-258-270	141-147-166	191-203-221	73-76-79	134-170-185	115-118-140	133-139-145	198-201
**Y6**	6	**P**	2002/2005	115-115-242	209-213-245	116-122-144	255-258-270	141-147-166	191-203-221	73-76-79	134-170-185	115-118-140	133-139-145	198-201
**Y7**	2	**P**	2002	115-115-244	209-213-245	116-122-144	255-258-270	141-147-166	191-203-221	73-76-79	134-170-185	115-118-140	133-139-145	198-201
**Y8**	1	**P**	2002	115-115-246	209-213-245	116-122-144	255-258-270	141-147-166	191-203-221	73-76-79	134-170-185	115-118-140	133-139-145	198-201
**Y9**	1	**P**	2002	115-115-248	209-213-245	116-122-144	255-258-270	141-147-166	191-203-221	73-76-79	134-170-185	115-118-140	133-139-145	198-201
**Y10**	2	**P**	2002	115-115-250	209-213-245	116-122-144	255-258-270	141-147-166	191-203-221	73-76-79	134-170-185	115-118-140	133-139-145	198-201
**Y11**	2	**P**	2002	115-115-254	209-213-245	116-122-144	255-258-270	141-147-166	191-203-221	73-76-79	134-170-185	115-118-140	133-139-145	198-201
**Y12**	3	**P**	2002	115-115-258	209-213-245	116-122-144	255-258-270	141-147-166	191-203-221	73-76-79	134-170-185	115-118-140	133-139-145	198-201
**Y13**	1	**P**	2002	115-115-262	209-213-245	116-122-144	255-258-270	141-147-166	191-203-221	73-76-79	134-170-185	115-118-140	133-139-145	198-201
**Y14**	3	**P**	2002/2005	115-115-264	209-213-245	116-122-144	255-258-270	141-147-166	191-203-221	73-76-79	134-170-185	115-118-140	133-139-145	198-201
**Y15**	3	**P**	2002	115-115-268	209-213-245	116-122-144	255-258-270	141-147-166	191-203-221	73-76-79	134-170-185	115-118-140	133-139-145	198-201
**Y16**	1	**P**	2005	115-115-242	201-209-245	116-122-144	255-258-270	141-147-166	191-203-221	73-76-79	134-170-185	115-118-140	133-139-145	198-201
**Y17**	1	**P**	2005	115-115-264	201-213-245	116-122-144	255-258-270	141-147-166	191-203-221	73-76-79	134-170-185	115-118-140	133-139-145	198-201
**Y18**	1	**P**	2005	115-115-246	209-213-251	116-122-144	255-258-270	141-147-166	191-203-221	73-76-79	134-170-185	115-118-140	133-139-145	198-201
**Y19**	1	**P**	2005	115-115-236	211-217-245	116-122-144	255-258-270	141-147-166	191-203-221	73-76-79	134-170-185	115-118-140	133-139-145	198-201
**Z1**	1	**P**	2005	115-115-*188*	209-233-245	*114*-*120*-144	255-*263*-*280*	147-*149*-166	191-203-221	73-76-79	134-170-*182*	115-115-140	133-139-145	198-201

Given are the genotype name, the number of individuals found to have this exact genotype, the site the genotypes were found (P – Río Purificación, G – Río Guayalejo), the year and the allele sizes (bp) for all 11 microsatellite loci. Alleles that are present in the triploid X lineage from G but not in the triploid Y and Z lineages in P are marked in bold typeface. Genotypes that differ between P and G even though all alleles are present in both populations are underlined. Alleles differing between the P lineages Y and Z are marked in italic typeface.

**Table 3:** Menken analyses of clonal diversity of the triploid lineages in the two sampling sites.

	**P 2002**	**P 2005**	**G 2002**	**G 2005**
**#ind**	26	13	11	15
**#clones**	15	8	4	3
**ENC**	12.52	6.26	2.20	2.42
**PDC**	0.58	0.62	0.36	0.20
**Unique**	7.00	5	1	0
**CD**	0.92	0.84	0.55	0.59
**CE**	0.78	0.78	0.55	0.81

For both years and sites the number of triploid individuals sampled (#ind), the number of different clones found within those individuals (#clones), the effective number of clones (ENC), the proportion distinguishable clones, the number of unique clones (unique), the clonal diversity (CD) and the clonal eveness (CE) are given.

**Figure 1:**
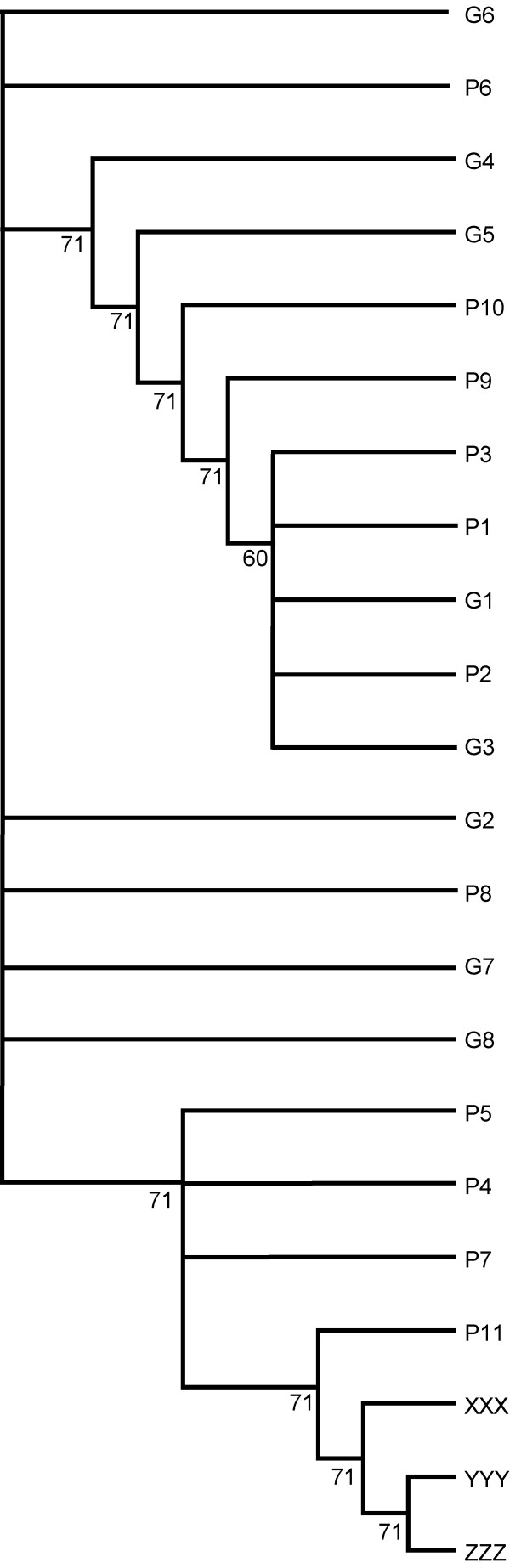
**Neighbour joining tree of individual genetic distances between diploid and triploid genotypes in P and G**. Shown are the triploid genotypes from P (YYY and ZZZ) and G (XXX) and several closely related diploid genotypes from both sites (P1–11; G1–8). Branches with a bootstrap support of less than 60% were collapsed. Bootstrapping support was derived from 1000 iterations.

In order to find out whether the triploids from the Guayalejo and from the Purificación system have a monophyletic origin, a phylogenetic tree using the Neighbour Joining method (NJ) method based on dmu^2 ^[17] was performed (Fig. 1). This revealed a clear differentiation between the X lineage found at G and the Y and Z lineages found at P.

**Figure 2:**
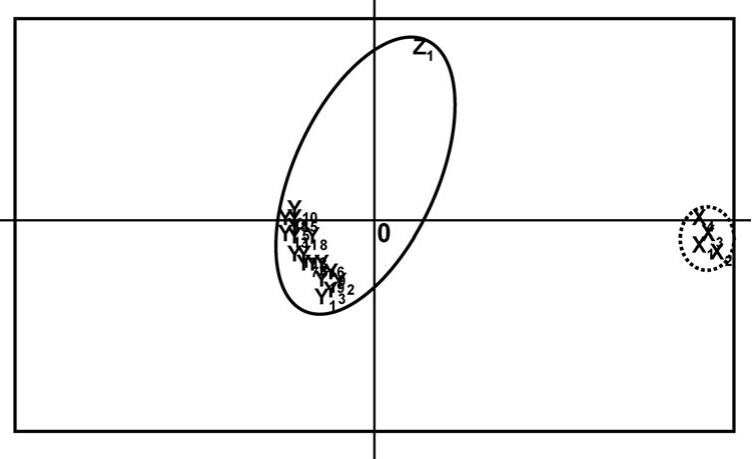
**Principal component analyses based on individual genotypes of the triploids found in the different sites**. The X axis is significant (P = 0.039) and explains 59% of the variability found in the data. The Y axis is not significant (P = 0.858). Levels of significance were derived from 10000 iterations. (Solid line – genotypes from P; dotted line – genotypes from G).

The separation of the triploids in G and P could also be shown in a principal component analysis: one significant axis (P = 0.039) was found that clearly separated the X genotype from the Y and Z genotypes and explained 59% of the variability found in the data (Fig. 2). A second axis separating the Z from the Y genotypes was found to be not significant (P = 0.858). A search for a closely related diploid genotype (meaning a diploid sharing all alleles with a triploid) for each triploid lineage revealed that one and the same diploid genotype was closest to all triploid lineages (X, Y, and Z). Such a diploid genotype was found at P (P11). This was confirmed by the neighbor joining tree (Fig. 1).

Mitochondrial DNA did not show any variability in the cytochrome b or the D-loop sequences analyzed. The same haplotype was found in the diploids as well as in the triploids from all sampling sites.

## Discussion

In this study we have analyzed a population of diploid and triploid *P. formosa *outside the Río Purificación system. From the high number of triploids found in both years (12% in 2002 and 16% in 2005) we conclude that in the Río Guayalejo system triploids actually are a stable part of the population. They were as common as in the formerly known Río Purificación field sites [12]. This is also in the range reported in the literature for the occurrence of triploids identified earlier at these and other sites [6,14,18].

Triploid lineages in this study were analyzed using 11 microsatellites as well as mtDNA. Mitochondrial DNA was found to be not variable enough to convey any information about the formation of the novel triploid lineage found in G. For all three investigated segments of the mitochondrial genome a single haplotype was present in diploid as well as triploid individuals from P and G. This may be due to the assumed single monophyletic origin of all diploid *P. formosa *[4,19,20].

Three microsatellites had been used in a former study to analyze clonal diversity of triploids at several sites of P and had uncovered only two different lineages: Y and Z [12]. Interestingly, two of the additional microsatellites used in this study proved to be very variable and showed that both triploid lineages show a certain degree of clonal variability. The high number of triploid genotypes detected in the P site is concordant with the findings from multilocus DNA fingerprints, which predicted a high genetic variability for triploids [12].

Triploids from the G site form a clearly distinct lineage and are less variable than triploids from the P site. The origin and the low diversity of the triploids in this site can be explained either by the hypothesis that an ancient population immigrated from G, diverged and then went through a bottleneck or that the triploids are of recent origin at the P site. Such an independent triploidisation at P seems to be the most likely explanation because our results show a clear separation of G (X) and P (Y+Z) genotypes in the NJ tree as well as in the principal component analyses. Even though triploids from G share several alleles with the triploids from P those alleles are very common in the diploid population and might therefore not have been inherited from a common ancestor.

Interestingly, the closest related diploid genotype to all three triploid lineages was found at P. This result was supported by the NJ tree. While diploid P and G genotypes were found to be mixed within separated branches the three diploid genotypes (P4, P7, P11) closest to the triploids clearly came from P. We hesitate to assume, solely based on this information, that the X genotype was derived from P because the frequency of diploid genotypes varies greatly between years and the diploid genotype P11 or the related true ancestral diploid genotype leading to the triploid lineage X might not have been collected so far at the P site or may no longer be around.

An explanation for the fact that the triploid lineages at the P and the G site are closest related to the same diploid genotype might be that successful triploidisation may only be possible in a certain diploid genotypic background, probably even a single clonal lineage. It would also fit the observation that the triploids, which occur quite frequently under laboratory conditions are always sterile [11] probably due to non-matching genotypes. In the hybridogenetic unisexual fish Poeciliopsis convincing evidence has been produced that triploid clones are generated de-novo quite frequently, but that the third genome was added always to the same allodiploid clone of a certain biotype [21].

A bottleneck due to breakdown of an old P derived triploid population (that invaded to G) and a recent recovery from a genotypically uniform small founder population is less likely because migration of a triploid genotype from P to G appears to be impossible due to geographic barriers: the G river system is the next south to the P river system but it is hydrogeographically completely separated by a high mountain range. In this context it is interesting to note that this border between the Río Purification/Río Soto la Marina system and the Guayalejo/Panuco river basin efficiently prevents transgression of fish species. It is the northern limit or *P. latipunctata *and *Xiphophorus variatus *and the southern limit of *X. xiphidium *[22].

We can of course not exclude that the triploid fish were artificially introduced into the Guayalejo system by human activities a long time ago, diverged here, and then from a genetic bottleneck re-established themselves from a highly divergent clone. River mouth hopping obviously happened in the spreading of diploid *P. formosa *[23] but is also unlikely as a source of triploids at G as triploids are not recorded despite extensive sampling from the lower Río Soto la Marina and the estuaries of both rivers. In addition, triploids apparently survive and are perpetuated only in the clear waters of upstream habitats [14].

Although the triploid lineage at G appears to represent an independent polyploidization event, this is only the second to be described in many samples analyzed to date. This is surprisingly rare compared to other asexual breeding complexes.

Multiple independent origins of gynogenetic triploid lineages have been found in the Poeciliopsis complex [24] and for the hybrid spined loaches of the genus Cobitis [25]. Here, multiple hybridization events of a diploid asexual fish with the host species established reproductive triploidy [25], leading to several independent triploid clonal lineages even at one and the same spot [24]. Also in the mixed ploidy unisexual breeding complexes of the Iberian minnow and the North-American genus Phoxinus, allotriploids are formed from diploid ancestors quite frequently [26,27].

Triploidy in general is very often connected to parthenogenesis. Parthenogenetic triploid systems are widespread and many examples are found amongst ants, lizards, salamanders, grasshoppers, fish, annelids, plathelminthes, nematodes, and plants. (For reviews see [28-32]). In those cases where the origin of the polyploid state is known to be due to "genome addition", it is a rather frequent event [28].

In summary, the triploids from the Río Purificación are according to our present knowledge all monophyletic derivatives of the same single introgression event [12]. On the basis of our analysis we favor the hypothesis that the triploids from the Guayalejo system arose from a second independent hybridization event of *P. formosa *with its sexual host species *P. mexicana*. It is also possible that the third genome is derived from *P. latipunctata*, which is a third host species for *P. formosa *and occurs in the Río Guayalejo and its tributaries [33]. However, this species has not been detected so far at the locality where the triploids were collected. It will be interesting to search at further sites in the future, whether other independent populations of triploid *P. formosa *exist.

## Conclusion

The microsatellite analysis identified the triploid fish form the Rio Guayalejo as being genetically divergent from the triploids of the Río Purificación. The most likely explanation is that these fish arose from independent paternal introgression events, through which an entire haploid genome of the gonochoristic host species was added to the non-recombining diploid genome of *P. formosa*. The fact that this introgression event was not a singular one, but might be much more frequent, supports the hypothesis that triploidy might be a relevant mechanism through which the new genetic variation is introduced. This can supplement the generally low genotypic diversity for adaptive changes to variable environments or parasites that clonal organisms suffer from [34,35]. In addition, accumulation of deleterious mutations as a result of the lack of recombination in an asexual organism could be at least partly compensated. It is, however, intriguing that reproductive triploidy occurs obviously extremely rare in *P. formosa*, compared to the other mode of paternal introgression, namely microchromosomes. Thus triploidy appears to be of less evolutionary importance for the long term survival of this asexual species.

## Methods

### Sampling

The collection site is a ford approximately 1 km southeast of the village of Emiliano Zapata at the Río Guayalejo (23°16.624'N 98°56.315'W). Individuals were captured using seine nets. One (5 mm^2^) piece of the fast regenerating dorsal fin was cut from each individual and stored in ethanol (70%) for transportation to the laboratory. Immediately after taking the biopsy, all animals were released. Samples were taken in February 2002 and in March 2005.

### Sample processing

All fins were cut in half to use one part to determine ploidy levels via flow cytometry and the second part to extract DNA for PCR analyses.

For flow cytometric measurements the fins were stained with the fluorescent dye DAPI, and ploidy levels were determined using chicken blood as a reference, essentially as described [36].

For microsatellite analysis DNA was extracted using 100 mM NaCl/0.5% Sarcosyl buffer and chelex (20%), according to [37]. The following primers were used: Sat1, KonD15, PR39 [12] and mCA16, mCA20, MSD23, mATG31, mATG38, mATG44, mATG61, mATG78 (originally from Walter et al 2004 [38] as reported in Lampert et al. 2006 [39]). From the primer pairs, one primer was Cy5 labelled (MWG, Ebersberg, Germany) at the 5' end, to allow detection by an ALF Express sequencer (Amersham Biosciences, Freiburg, Germany) (see below). PCR reactions: 300 sec of denaturing, 30 sec at 94°C, 30 sec at annealing temperature (52°C for KonD15/58°C for Sat1/55°C for all other primers) and 30 sec at 72°C for 35 cycles followed by a 300 sec elongation step. PCR products were analyzed on an ALF Express sequencer using an external marker (50–500 bp in 50 bp steps) (Amersham Biosciences, Freiburg). Internal standards were used to facilitate fragment alignment. ALF Express created files (.alx) were converted into alf files using the appropriate program ALX2ALF. .alf files were analyzed via the FM (Fragment Manager) program (Amersham Biosciences, Freiburg).

To confirm the morphological identification of the fish as either *P. formosa *or *P. mexicana *a diagnosis by PCR with primers for the *src *gene was performed [20]. Primers ScrE and ScrF give one product of approximately 650 bp from *P. mexicana *DNA and two products of 650 bp and 700 bp from *P. formosa*. Homo- or heterozygosity of individuals and hence species identity can easily be determined on 2% agarose gels.

Extracted DNA was also used to investigate variability of mitochondrial DNA (mtDNA) in the triploids found in both sites. We sequenced two parts of the cytochrome b gene and parts of the D-loop sequence in triploids and in two diploids from the two sites (P and G). The diploid genotypes used for the comparison of mtDNA genes were picked due to their very different microsatellite allelic pattern. For cytochrome b we used the primers L14724/H15149 and L15513/H16100 and for the D-loop primer pairs L15995/H16498 were used (all from Meyer et al. 2004 [40]). Amplification was done using the PCR conditions recommended by Meyer et al. We directly sequenced the PCR products using the appropriate H and L PCR primers. Sequencing protocol using the DTSC method followed the Beckman-Coulter instructions. Sequences were analyzed using a Beckman-Coulter capillary sequencer and the Ceq8000 software provided by the company. Sequence alignments were done using the BioEdit program [41].

### Data analyses

Triploid genotypes found at the Río Guayalejo site (G) were compared to the triploid genotypes from the Río Purificación site (P) from the same years for levels of allelic and genotypic variability. Only individuals that could be genotyped at all 11 microsatellite loci were included in the statistical analyses. Genetic diversity among triploids was described following Menken et al. (1995) [16]: the proportion of distinguishable clones (pdc = number of clones divided by sample size; a value of 1.00 means every individual has a different genotype), the effective number of clones (enc = 1/∑*p*_*i*_^2^, *p*_*i *_– frequency of the *i*th clone in a population), clonal diversity (cd = 1-∑*p*_*i*_^2^) and clonal evenness (ce = (1/∑*p*_*i*_^2^)/Nc – Nc number of clones (Clonal evenness (ce) gives a relative and therefore comparable measure for the distribution of clones. A ce value of one means all clones are equally distributed.) were calculated.

We used the program POPULATIONS [42] to calculate individual distances based on Goldstein's dmu^2 ^[17] between genotypes. To enable the inclusion of diploid genotypes and to avoid an "oversampling" of the Y branch only three microsatellite loci (Sat1, KonD15, PR39) were included in the calculations. Individual distances between genotypes were used to create a neighbor-joining (NJ) tree. Bootstrapping values for the NJ tree were obtained from 1000 iterations. In addition, we performed a principal component analysis based on all 11 microsatellite loci using the program PCAGen [43] of individual genotypes. 10.000 iterations were used to obtain p-values for the axes found. As the program PCAGen only accepts diploid genotypes we re-coded the triploid genotypes that were based on 11 microsatellite loci as 16 diploid loci treating the alleles as independent units (e.g. 010203 040506 (two triploid loci) => 0102 0304 0506 (three diploid loci). Results of the analysis did not change if loci were re-coded in a different order (e.g. starting from the last locus or random).

## Competing interests

The author(s) declare that they have no competing interests.

## Authors' contributions

SS carried out part of the molecular genetic and cytological analyses. KPL participated in the field data collection, carried out part of the molecular genetic and cytological analyses, performed the statistical analyses and drafted the manuscript. DKL participated in the field data collection and coordination of the study. FJGL participated in the field data collection and coordination of the study. MS conceived the study, designed and participated in its coordination, organized and participated in the field study, and drafted the manuscript. All authors read and approved the final manuscript.
